# HELPING OTHERS IS A MIXED BLESSING: IMPLICATIONS FOR DAILY WELL-BEING

**DOI:** 10.1093/geroni/igz038.875

**Published:** 2019-11-08

**Authors:** Meng Huo, Yee Ng, Karen Fingerman

**Affiliations:** 1 The University of Texas at Austin, Austin, Texas, United States; 2 University of Texas at austin, Austin, Texas, United States

## Abstract

The literature documents mixed findings regarding how helping others influences individuals’ mental and physical health. We assessed various types of support that older adults offered (e.g., emotional, practical, advice) and examined how helping others was associated with older adults’ daily mood and physical activity. This study utilized data from the Daily Experiences and Well-being Study, where 293 participants aged 65+ reported on their helping behaviors and mood at the end of each day across 5 days. Participants also wore Actical accelerometers to track physical activity. Multilevel models revealed that older adults reported greater negative mood and less physical activity on days when they provided emotional support. Yet, giving advice was associated with increased positive mood that day. Moreover, older adults spent less time being sedentary on days when they offered practical help. This study offers insights into psychological and health consequences of helping others by examining older adults’ everyday lives.

